# *MET* exon 14 skipping is overexpressed in an allele-specific manner in lung adenocarcinoma primary samples

**DOI:** 10.17912/micropub.biology.000957

**Published:** 2023-09-26

**Authors:** Megan Durham, Swetha Vadde, Angela N Brooks

**Affiliations:** 1 Department of Molecular, Cell and Developmental Biology, University of California, Santa Cruz, Santa Cruz, California, United States; 2 Department of Biomolecular Engineering, University of California, Santa Cruz, Santa Cruz, California, United States

## Abstract

*MET* exon 14 skipping ( *METΔ14* ) is a well-characterized oncogene in the Ras-MAPK pathway driving lung adenocarcinoma (LUAD). Previous studies on *METΔ14* revealed this aberrantly spliced oncogene is expressed in LUAD primary samples and is associated with heterozygous somatic mutations and deletions near exon 14 splice sites. Upon further examination of DNA and RNA sequencing data from primary samples, we highlight that *METΔ14* is overexpressed in an allele-specific manner. These data suggest that dose-dependence of *METΔ14* may be critical to oncogenesis.

**Figure 1. Allele-specific overexpression of METΔ14 in Lung Adenocarcinoma primary samples:**
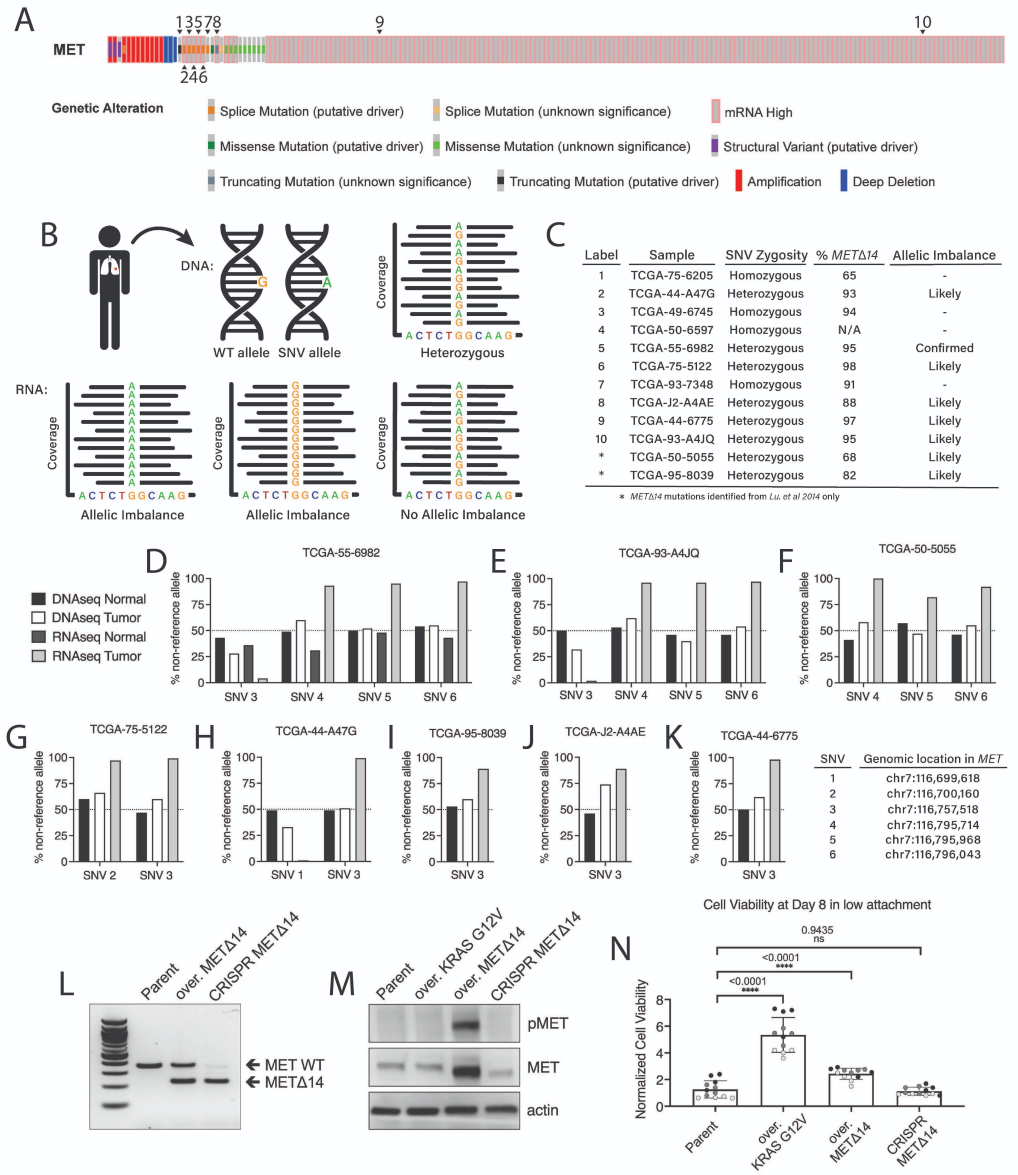
A) Oncoprint adapted from cBioPortal reveals genetic mutations in *MET* per LUAD TCGA primary sample. Arrows correspond to TCGA IDs in C from (Lu et al., 2017). Note not all splice mutations in *MET* are captured using the analysis from cBioPortal. High *MET* expression is relative to normal samples. B) Diagram describing how allelic imbalance was identified in TCGA DNA and matched RNA sequencing data. At the DNA level, lung adenocarcinoma tumors heterozygous for germline SNVs within the *MET* gene were identified and chosen for further analysis. These SNV locations were identified in matched RNA-seq data, and ratios of SNV to WT nucleotide determined if there is an allele-based expression of *METΔ14* . C) Table summary of TCGA sample, SNV zygosity from DNA-seq data, allelic imbalance from RNA-seq data, and percent *METΔ14* expression calculated with JuncBase results from (Soulette et al., 2023). D-K) Graphs representing the percentage of non-reference allele for heterozygous germline SNVs in TCGA primary samples from C. Genomic coordinates are relative to hg38. L) RT-PCR using primers spanning exon 14 of *MET* to validate *METΔ14* mRNA in an overexpression AALE line (over. METΔ14) and CRISPR-generated AALE cells (CRISPR METΔ14). M) Western blot confirming activated and total MET in an overexpressed KRAS G12V AALE cell line (over. KRAS G12V), over. METΔ14 and CRISPR METΔ14. N) Growth in low attachment (GILA) cell viability assay of 3 biological replicates of over. KRAS G12V, over. METΔ14 and CRISPR METΔ14. Colors group four technical replicates. Comparisons performed with a Mann-Whitney test. Error bars represent standard deviation of the mean.

## Description

MET is a tyrosine kinase receptor in the Ras-MAPK pathway whose unchecked signaling leads to cancer. This receptor is activated through binding of its ligand, HGF (Bottaro & Rubin, 1991) , and this signal is terminated through MET’s ubiquitination and degradation (Jeffers et al., 1997) . The negative regulatory region of MET is encoded by exon 14, and contains a binding site for the E3-ubiquitin ligase, Cbl, at position Y1003 within the cytoplasmic juxtamembrane domain (Peschard et al., 2001) . Phosphorylation of Y1003 permits Cbl binding, triggering MET’s ubiquitination and subsequent degradation (Peschard et al., 2001) . The inclusion of exon 14 is critical for MET’s regulation and mutations at or near exon 14 splice sites are associated with exon exclusion from the mature mRNA ( *METΔ14* ) (Lu et al., 2017) . This exclusion results in a protein lacking the Cbl binding site, extending the protein's life span in the membrane, and prolonging proliferative signaling that drives cancer (Kong-Beltran et al., 2006; Lu et al., 2017; Ma et al., 2003, 2005) . These *METΔ14* mutations are observed in 2.8% of cases of LUADs (Lu et al., 2017) , at a relatively high frequency, considering *MET* is estimated to be mutated in 4-6% of lung adenocarcinomas (Caso et al., 2020) . These data suggest that nearly half of all *MET* mutations are splicing mutations.

Two separate studies investigating *METΔ14* revealed that although both primary samples and cell lines were heterozygous for exon 14 deletions, predominantly, the *METΔ14* isoform was produced (Kong-Beltran et al., 2006; Onozato et al., 2009) . Furthermore, Lu *et al.* identified somatic mutations at exon 14 of *MET* in a larger cohort of LUAD samples from The Cancer Genome Atlas (TCGA) and found that the majority of these samples overexpress *MET* and predominantly express the mutant isoform (Lu et al., 2017) . These three studies suggest an allele-specific expression bias of *METΔ14* . Allele-specific expression occurs when one allele exhibits a higher level of expression compared to the other and is implicated in cancer (Bielski et al., 2018; Castel et al., 2018; Mayba et al., 2014; Ongen et al., 2014; PCAWG Transcriptome Core Group et al., 2020) . Notably, this bias in allele expression can directly influence the expression of cancer driver genes through specific expression of the oncogenic allele (Bielski et al., 2018; Mayba et al., 2014) . We hypothesized that *METΔ14* undergoes allele-specific expression which may be critical to its transformative abilities. The *Lu et al.* study did not further investigate the zygosity of the LUAD samples nor any associated copy number changes, which may contribute to allelic imbalance. Additionally, the matched DNA and RNA sequencing data associated with these samples permit tracking of allele usage. Therefore, we further investigated the underlying mechanism driving the predominant expression of *METΔ14* in these LUAD samples.

While typically allele-expression bias is due to copy number alteration (PCAWG Transcriptome Core Group et al., 2020) , we found no sample with a *METΔ14* mutation and an associated copy number amplification of the *MET* gene ( Figure 1A ). This is consistent with previous findings revealing that *METΔ14* and *MET* copy number alterations are mutually exclusive (Baldacci et al., 2020; Guo et al., 2019; Onozato et al., 2009) . However, the majority of *METΔ14* samples also have co-occurring *MET* mRNA overexpression ( Figure 1A ). This indicates these *METΔ14* samples employ a different mechanism to drive *MET* overexpression independent of MET copy number alterations.

To determine if zygosity could explain *METΔ14* allele-specific expression, we compared whole exome and matched RNA-seq data from these TCGA samples ( Figure 1B and 1C). Additionally, we confirmed the strong bias of *METΔ14* mutant allele expression from the quantification of the percentage of *METΔ14* isoform usage in the LUAD tumor samples (Soulette et al., 2023) ( Figure 1C ). We used germline single nucleotide variants (SNVs) to determine both zygosity and to track which allele was used to express *MET.* We manually scanned the entire length of the *MET* gene for evidence of SNVs. For heterozygous samples, we identified the ratio of RNA expression at the SNVs to determine the allelic imbalance in these samples. ( Figure 1B ). To confirm this allele-specific expression of *METΔ14* is unique to cancer, it is necessary to analyze both the tumor and matched normal samples; however, we identified only one *METΔ14* sample that was heterozygous and had a matched normal sample ( Figure 1D ). As predicted, while the matched normal sample equally expressed both the wild type and non-reference SNVs, there is a clear allele-specific expression in the tumor. While the remaining heterozygous samples lacked matched normal RNA-seq data, all samples exhibited an allelic imbalance in the tumor ( Figure 1E -K). This suggests the allele-specific expression of *METΔ14* is a widespread phenomenon among these LUAD tumors. Furthermore, given that 8/12 of *METΔ14* samples also overexpress total *MET* compared to normal samples ( Figure 1A ), this suggests that *METΔ14* is overexpressed in an allele-specific manner in these samples.

In a study of cancer genomes examining the positive selection of oncogenic driver mutant alleles through ploidy changes, alleles expressing activating mutations in *MET* were found to experience strong positive selection (Bielski et al., 2018) . This study examined all *MET* oncogenic driver mutations at DNA-level copy number selection, which does not capture RNA-level changes in expression. However, the work presented in our publication uses both DNA and RNA sequencing data to show that ploidy changes are not required for allele-specific expression of *METΔ14* ( Figure 1A ). Given the consistency of *METΔ14* overexpression in the majority of samples examined, our data suggest the overexpression of the oncogenic allele is required for *METΔ14* -driven cancer progression. Further studies are necessary to understand the molecular mechanism of the allele-specific overexpression which could involve *cis* -acting genetic or epigenetic factors, or allele-specific transcript stability.

We believe that two factors drive *METΔ14* as an oncogene in LUAD: 1) *METΔ14* allele-specific overexpression, and 2) the abundance of MET ligand, HGF (Lu et al., 2017) . These factors have implications for how *METΔ14* is studied. A body of work aims to decouple the *METΔ14* mutation from overexpression due to *MET* amplification by using CRISPR-based methods (Lu et al., 2017; Togashi et al., 2015; Wang et al., 2022) . With these methods, mutations are created at splice sites of exon 14 to force the production of *METΔ14* from the endogenous promoter to transform cells in the presence of its activating ligand, HGF. However this CRISPR-based expression does not lead to overexpression of the *METΔ14* mRNA. We compared the ability of overexpressed and CRISPR-based *METΔ14* to provide immortalized tracheobronchial epithelial cells (AALE) (Lundberg et al., 2002) a proliferative advantage in a growth in low attachment (GILA) assay (Rotem et al., 2015) . While we confirmed CRISPR-based *METΔ14* mRNA ( Figure 1L ), this did not induce activated MET protein ( Figure 1M ). Furthermore, only overexpressed *METΔ14* confers a selective advantage in the GILA assay ( Figure 1N ). This activation occurs independent of HGF, revealing the oncogenic potential of *METΔ14* overexpression alone. These experiments provide further evidence that MET exon 14 skipping, alone, is not sufficient for oncogene activation and that there needs additional increased dosage of the METΔ14 allele; consistent with the characterization in primary samples. Thus, we suggest future studies to functionally characterize *METΔ14* will more accurately recapitulate tumor cells if *METΔ14* is overexpressed and performed with and without the presence of ligand.

## Methods

*Data Acquisition*

For each TCGA sample from the Lung Adenocarcinoma TCGA PanCancer Atlas cohort labeled in Figure 1C, previously -aligned whole exome (DNA-seq) and RNA-seq samples were securely downloaded from the NCI Genomic Data Commons. The OncoPrint was generated in cBioPortal (Cerami et al., 2012; Gao et al., 2013)

*SNV Characterization*

All TCGA samples were associated with files: tumor and matched normal DNA-seq data, as well as tumor RNA-seq data. Two TCGA samples had matched normal RNA-seq data, which could be used to confirm cancer-specific expression. To visualize allele-specific expression within this data, all files per sample were imported into Interactive Genomics Viewer (IGV) (Robinson et al., 2011) . As SNVs provide a track record of allele abundance, we scanned the entire MET gene and determined relative percentages of SNVs at those loci between the DNA and RNA-seq data, which can be calculated in IGV. For the DNA-seq, a near 1:1 ratio of SNV to wild type allele indicates heterozygosity. For the matched RNA-seq data, a proportion of SNV to wild type allele close to 0% or 100% of heterozygous SNV loci indicates allelic imbalance.

*Cell line generation*

AALE cell lines (Lundberg et al., 2002) were cultured in SAGM Small Airway Epithelial Cell Growth Medium BulletKit (CC-3118, Lonza) using the ReagentPack Subculture Reagents (CC-5034, Lonza). Cells were grown under constant 37°C and 5% CO _2_ . CRISPR-expressed lines were generated by transfecting AALEs with stable Cas9 expression, and using the below single guide RNA (sgRNA) expressed with a lentiviral vector targeting the splice site of *MET* exon 14:

METsg8: 5’- TACCGAGCTACTTTTCCAGA -3’

Overexpressed lines were generated using lentiviral plx317 *METΔ14* and plx301 KRAS G12V, a gift from Alice Berger.

*RT-PCR METΔ14 mRNA Validation*

RNA was isolated via TriReagent (Sigma-Aldrich, T9424) and the Zymo Direct Zol RNA Miniprep kit (Zymo Research Corporation, R2050). Subsequent cDNA prep was performed using the High-Capacity cDNA Reverse Transcription Kit (Thermo Fisher Scientific, 4368814), and resulting cDNA was used as a PCR template using the HotStart ReadyMix PCR Kit (Kapa Biosystems, KK6202). Exon spanning primers were ordered from IDT:

MET_splice_F: 5’- TGGGTTTTTCCTGTGGCTGA -3’

MET_splice_R: 5’- GGGCCCAATCACTACATGCT -3’

and run using these cycling conditions: initial denaturation of 95°C 3min, 30 cycles of 98°C 20sec, 61°C 15sec and 72°C 30sec, a final extension of 72°C 2min.

Resulting amplicons were run on a 1.2-2% agarose gel until wild type and exon-skipped isoforms were resolved. Relative isoform abundance ratios were quantified using Image Studio Lite.

*Western Blot*

One tablet of Protease Inhibitor cocktail (Roche, 04693124001) was added to a 10mL aliquot of RIPA lysis buffer (Thermo Fisher Scientific, 89900) and dissolved completely. Cells were grown to 80% confluency in 10cm tissue treated dishes (Santa Cruz Biotechnology, Inc., sc-200286). Plates washed 2x with ice cold DPBS (Life Technologies Corp., 14190144). 1mL ice cold RIPA with Protease Inhibitor was added to the cells and scraped into 2mL tube. Tubes were incubated on ice and vortexed periodically. Lysed cells were pelleted at max speed in a chilled centrifuge for 10min, then supernatant was aliquoted in volumes of 200μl. Lysates were stored at -80°C.

Lysate was sonicated at the maximum setting for increments of 30 seconds 2x. Lysate was left to recover on ice for 1 minute between sonications. The protein content in the sonicated samples was quantified using the Pierce BCA Protein Assay Kit (Thermo Fisher Scientific, 23225).

30μg protein was added to 11.7μl MLB (1:10 dilution of 2-mercaptoethanol (Bio-Rad, 1610710) and 4x Laemmli sample buffer (Bio-Rad, 1610747)), and volume was brought to 46.7ul total using RIPA buffer in a 2mL tube. Samples were denatured at 95°C for 5 minutes. Samples and Precision Plus protein standards (Bio-Rad, 1610374) were loaded into a 4-15% Mini-PROTEAN TGX Precast Gel (Bio-Rad, 4561083EDU). Gel was run at 70V using 1x TGX Buffer (Bio-Rad, 1610772) until adequate separation of ladder was obtained.

The Trans-Blot Turbo RTA Transfer PVDF kit (Bio-Rad, 1704272) protocol with 1x Bio-Rad Transfer Buffer (Bio-Rad, 10026938) was used with the High Molecular Weight setting. After the transfer, the membrane was blocked with 4% BSA (Millipore Sigma, A3059) for 1 hour. Then 1:2000 primary antibody (pMET (Cell Signaling Technology, 3077), total MET (Cell Signaling Technology, 8198) was incubated at 4°C overnight.

The next day, the membrane was washed 3x 10min with 1x TBST (1x TBS with 1mL Tween-20 (Fisher Scientific, BP337)). Blots were incubated with 1:1000 HRP-conjugated secondary antibody (Li-Cor, 92601000) in 4% BSA for 1 hour. Blots were washed 3x 5min in 1x TBST. Luminol pen (Li-Cor, 926-91000) was used to mark ladder, then blots were incubated with ECL (WesternSure PREMIUM Chemiluminescent Substrate (Li-Cor, 926-95000) for 5 minutes. Blots were visualized with C-Digit Imager (Li-Cor) according to equipment instructions.

To blot for actin, blots were washed with 1x TBST 3x 10min, then incubated in 1:1000 actin-HRP antibody (Cell Signaling Technology, 7074S) in 4% BSA. Blots were washed 3x 5min in 1x TBST, ladder marked with Luminol pen, incubated with ECL for 5 minutes, and imaged as above.

*GILA Cell Viability Assay* AALE cells were grown to ~80% confluency. Plates were trypsinized and 2,500 cells in a volume of 100ul were seeded per well into a 96 well low attachment plate (Corning, 3474). Four technical replicates were used per cell line. Additionally 2,500 cells per well (100ul) was seeded into a flat bottom 96 well assay plate (Thomas Scientific, 290-8027-W1F). It's important to leave one well of space in between each replicate, as to avoid fluorescence bleed over during measurement. The low attachment plate was left in a 37°C incubator for 8 days. Cell viability in the assay plate, or the ‘Day 0’ measurement, was assessed in order to normalize Day 8 to the initial plating concentration. First, Cell Titer Glo (Promega, G7572) was allowed to come to room temperature. Then, a 1:1 ratio of Cell Titer Glo was added to the cells using a multichannel pipette, so the total volume was 200ul. Plates were shaken 2 minutes by hand to lyse the cells, then mixed with single channel pipette by pipetting 4 -5 times. The plate was left undisturbed and sheltered from light for 20 minutes, then emitted fluorescence was measured using the Varioskan LUX microplate reader (Thermo Fisher, VL0000D0). This provided raw values of fluorescence proportional to the amount of ATP produced, and served as a normalization value for Day 0 per cell line. After 8 days, both Cell Titer Glo and the low attachment plate were allowed to come to room temperature, and lysis and imaging protocol repeated as above. These raw values were normalized to the average of the four Day 0 replicates to compare cell viability between cell lines.
